# Commonly Used Subjective Effort Scales May Not Predict Directly Measured Physical Workloads and Fatigue in Hispanic Farmworkers

**DOI:** 10.3390/ijerph20042809

**Published:** 2023-02-05

**Authors:** Ornwipa Thamsuwan, Kit Galvin, Pablo Palmandez, Peter W. Johnson

**Affiliations:** 1 Department of Mechanical Engineering, École de Technologie Supérieure, Montreal, QC H3C 1K3, Canada; 2 Department of Environment and Occupational Health Sciences, University of Washington, Seattle, WA 98105, USA

**Keywords:** correlation, Borg, metabolic load, percent of heart rate reserve, muscle fatigue, electromyography

## Abstract

In North America, Hispanic migrant farmworkers are being exposed to occupational ergonomic risks. Due to cultural differences in the perception and reporting of effort and pain, it was unknown whether standardized subjective ergonomic assessment tools could accurately estimate the directly measured their physical effort. This study investigated whether the subjective scales widely used in exercise physiology were associated with the direct measures of metabolic load and muscle fatigue in this population. Twenty-four migrant apple harvesters participated in this study. The Borg RPE in Spanish and the Omni RPE with pictures of tree-fruit harvesters were used for assessing overall effort at four time points during a full-day 8-h work shift. The Borg CR10 was used for assessing local discomfort at the shoulders. To determine whether there were associations between the subjective and direct measures of overall exertion measures, we conducted linear regressions of the percentage of heart rate reserve (% HRR) on the Borg RPE and Omni RPE. In terms of local discomfort, the median power frequency (MPF) of trapezius electromyography (EMG) was used for representing muscle fatigue. Then full-day measurements of muscle fatigue were regressed on the Borg CR10 changes from the beginning to the end of the work shift. The Omni RPE were found to be correlated with the % HRR. In addition, the Borg RPE were correlated to the % HRR after the break but not after the work. These scales might be useful for certain situations. In terms of local discomfort, the Borg CR10 were not correlated with the MPF of EMG and, therefore, could not replace direct measurement.

## 1. Introduction

In the past several decades, the majority of farmworkers in the United States and Canada are Hispanic migrants and more than half of the population do not have authorization to work in the United States [1]. Despite being essential to the North American economy, language, cultural, immigration status, and educational barriers can negatively affect the migrant farmworkers’ ability to communicate and/or characterize their exposure to physical workplace hazards. Compared to US-born farmworkers, undocumented Hispanic workers have more precarious work, earn less, and are more often paid a piece rate than hourly [2]. This situation has led to more exposure to physical and mental stress [3,4], ultimately contributing to work-related musculoskeletal injuries [5,6]. Notably, according to a study of physician examinations in the United States, there was a 35% prevalence of work-related musculoskeletal disorders among Latino migrant farmworkers [7]. Moreover, Latino migrant farmworkers have been facing language barriers [8] which have impacted their levels of risk in terms of occupational health and safety [9]. Furthermore, they have been unlikely to report their poor working conditions [10]. Additionally, these known problems have been affected by increasing age and being female [6,11] as well as being compensated at a piece rate, which could incentivize them to work harder and avoid taking frequent or long breaks [12].

Field ergonomic assessments can be conducted by direct measurement with sensors and by questionnaires with subjective self-reported ratings. Various available subjective ergonomic assessment tools such as the RULA, REBA, Borg RPE, Borg CR-10, and standardized Nordic questionnaires have been used to assess the ergonomic risk factors among other agricultural worker populations [13]. However, the development and validation of subjective work assessment tools for this population has been lacking.

Despite many laboratory validation studies of subjective scales, studies on the associations between direct and subjectively reported ergonomic measures are lacking in the field settings. It is unclear whether the subjective rating scales validated in the controlled environment could be applied to ergonomic assessments in the field.

In the context of Hispanic (both migrant and non-migrant) farmworkers in North America, the primary objective of this study was to determine whether the subjective measures of the exertion of the overall body and at the local body parts could be used to predict the workload and fatigue directly measured with sensors. Specifically, this study aimed to determine: (1) the association between metabolic, i.e., cardiovascular, load and the overall Omni RPE and Borg RPE scales; and (2) the association between localized muscle fatigue measured through electromyography and the local Borg CR10 scales.

## 2. Methods

### 2.1. Participants

The participants were selected to represent the major characteristics of Hispanic male farmworkers in an industrialized orchard in the state of Washington, as well as in the United States in general. The research team members include male and female local researchers of Latin American origin, who understand the cultural dynamics within the population. The orchard owners and managers were initially contacted for permission to conduct research at their site. Then all participants were recruited on the day prior to the first day of data collection. A total of 24 farmworkers participated in this study. The participants were equally divided into three groups, i.e., eight different farmworkers per group, based on three different harvesting methods: (1) picking apples at the lower level of the trees up to their reach distance overhead, denoted as “Ground” workers; (2) using a ladder to pick apples from the whole trees, denoted as “Ladder” workers; and (3) picking apples at the upper level of the trees while standing on a semi-automated mobile orchard platform, denoted as “Platform” workers. All the participants worked to the same schedule, from 7:30 to 15:00 with a break between 9:00 and 10:30.

### 2.2. Ergonomic Exposure Parameter Selection

#### 2.2.1. Overall Exertion

The metabolic equivalent (MET), defined as the oxygen consumption of a person and representing the rate at which a person burns energy, has been widely accepted as a measure for the intensity of a physical exercise or work among adults aged 18 to 65 years old [14,15]. Since it is difficult to measure oxygen consumption outside laboratories, numerous studies have estimated the MET using other measures that are feasible in the field, such as acceleration [16,17] and heart rate [17,18,19]. In occupational health, a heart-rate-based approach was also developed for determining the cost effectiveness of ergonomic interventions, including break strategies (i.e., duration and frequency), as well as the provision of air-conditioning [20]. In agricultural field settings, heart rate has been used for evaluating the work conditions in several activities, such as cultivating potatoes [21], rice [22], wheat [23], and apples [24]. However, one drawback with heart rate-based methods is that heart rate varies according to age, sex, BMI, and fitness level [25]. For the aforementioned reasons, percentage of heart rate reserve (% HRR), which adjusts for the maximum heart rate and resting heart rate and has a strong association with oxygen uptake [26], has been proposed to be a predictor of MET. % HRR is a measure of the heart’s ability to recover to a resting state after physical activities. % HRR was validated as a proxy for physical exertion in comparison to oxygen uptake in laboratory settings for cardio exercises [27] as well as during resting and sleeping [28] On farms, % HRR has been used for evaluating an assistive device for digging tasks [29].

#### 2.2.2. Local Body Fatigue

Surface electromyography (EMG) has been used for assessing muscle activity in the field of occupational ergonomics [30,31]. The EMG amplitude has a dose–response relationship with the force applied during muscle contraction [30]. Furthermore, based on the spectral analysis of an EMG signal, a decrease in the EMG mean power frequency (MPF) has been shown to be associated with muscle fatigue [31]. Surface EMG has already been used for evaluating physical work demand, and for comparing new and traditional harvesting tools. In laboratory studies, surface EMG has been used for evaluating the efficacy of emerging technology such as vertical keyboards [32] and the size of different touch screen keyboards [33,34]. Nowadays, it is feasible to apply EMG techniques in field settings for general ergonomic assessments and to follow up on effectiveness after the implementation of technology. In construction, EMG-based muscle fatigue has been used in assessing scaffold building activity [35]. In forestry, surface EMG has also been used for measuring muscle activity at the trapezius of machine operators [36]. In different agricultural fields, EMG has been used to assess the on-farm exposure to biomechanical risk factors [37], evaluate the efficacy of a rotary milking parlor compared to herringbone and parallel milking parlors [38], compare the differences between farmworkers using ladders and a mobile orchard platform in the fruit picking industry [39], and investigate the potential uses of an exoskeleton or a personal assistive suit for selected manual farm tasks [29,40].

#### 2.2.3. Subjective Measures

On the one hand, the Borg RPE [41], which is a perceived exertion scale ranging from 6 to 20, has been widely considered as a psychosomatic indicator of physical activity intensity during work. The Borg RPE was developed in the context of cardiovascular treadmill exercise and intended to be highly correlated with heart rate; that is, the RPE score multiplied by 10 generally represents a person’s actual heart rate in beats per minutes.

On the other hand, the Borg CR10 [42] which is anchored at a number scale from 0 to 10, has been used as a local pain scale. The Borg CR10 has applications in ergonomic assessments, for instance, as a predictor of grip forces during hand-tool tasks tested in a laboratory setting [43] and as an indicator of the risks of injury among janitors [44] and nurses [45]. One disadvantage of the Borg CR10 is a potential error due to its subjective nature, thus, it is required to calibrate for the maximal level of exertion to achieve high accuracy [46].

The Borg RPE and CR10 scales have been translated into several languages [47,48,49]. While the Borg scales have a verbal description for each numeric value, another commonly used and validated scale, the Omni RPE, includes pictorial descriptors specific to the context, along with anchor words. Thus, the Omni RPE is thought to be more generalizable [50].

The relationships between subjectively reported and direct measures have been investigated in laboratory studies. A previous study found a significant linear relationship between the EMG MPF of the upper trapezius and the Borg CR10 results for shoulder elevation endurance tasks [51]. Similarly, correlations were found between the EMG MPF of the lumbar muscle and the Borg CR10 results during repetitive and prolonged trunk extension tasks [52,53]. Regarding muscle activity, i.e., the amplitude of the EMG signal, another study [54] observed that there was a relationship between the EMG amplitudes in the percentage of maximum voluntary contraction and the subjective ratings of discomfort [55] in hand tool use, for the trapezius only, but not for other muscles.

### 2.3. Data Collection

#### 2.3.1. Heart Rate Monitors

The participants’ heart rates in beats per minute were sampled every second throughout a full work day using a heart monitor (Polar RS100CX; Polar Electro Inc., Lake Success, NY, USA).

#### 2.3.2. Electromyography

A local muscle activity signal was collected from both the left and right trapezius muscles at 1000 Hz using single-use disposable 10 mm diameter pre-gelled electrodes (Blue Sensor N; Ambu, Ballerup, Denmark), with a 20 mm inter-electrode spacing. The differential electrode pairs were placed 1cm distally from the midpoint between the C7 of the spinal column and the acromion, and the ground electrodes were placed on the acromion. The electrodes were connected with pre-amplifier wires to a battery-powered portable data logger (Biomonitor ME6000; Mega Electronics Ltd., Kuopio, Finland).

#### 2.3.3. Subjective Ratings: Borg RPE, Omni RPE, and Borg CR10

**Figure 1:**
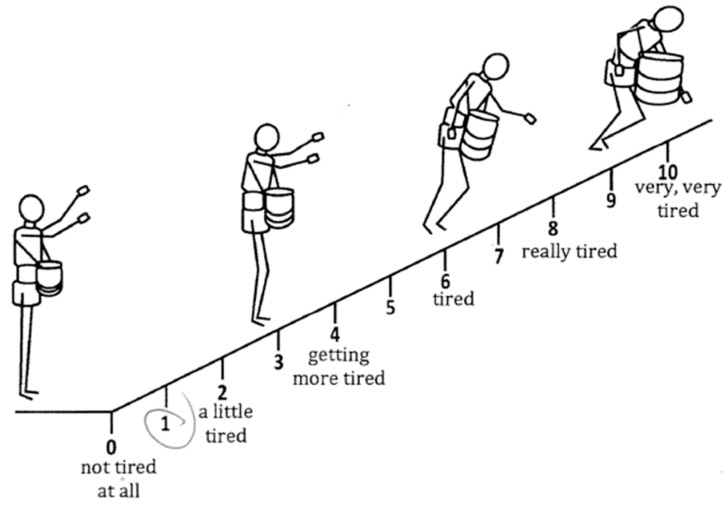
Omni RPE used in this study.

Borg the RPE and Omni RPE scales were used as subjective measures of the overall effort exerted by the workers, and the Borg CR10 scale, which asked the participants questions such as, “How tired does your right shoulder feel?”, was used as a subjective measure of local discomfort, particularly in terms of the level of tiredness (“cansado” in Spanish) they felt at each specific body part at that moment. The Borg RPE scale, ranging from 6 to 20, was accompanied by verbal anchors, from “no exertion” at 6, to “maximal exertion” at 20. The Borg CR10 was also accompanied by verbal anchors, from “not tired” at 0, to “severely tired” at 10. The Spanish version of the Borg RPE and Borg CR10 have previously been validated in the field [24]. In addition, the Omni RPE, with pictures of humans wearing an apple bag, was included (Figure 1). All the self-report survey instruments were administered individually to the participants by fluent Spanish-speaking team members. The participants could view the questions while the team member read them aloud.

The measurement time points of the effort surveys were:

T0: before starting the work shift (15-min heart rate measurement)

T1: after working for 90 min since the beginning of the work shift (10-min heart rate measurement)

T2: after taking a break for 30 min, immediately after the 90-min work period (10-min heart rate measurement)

T3: at the end of the 7.5-h work shift (10-min heart rate measurement)

**Figure 2:**
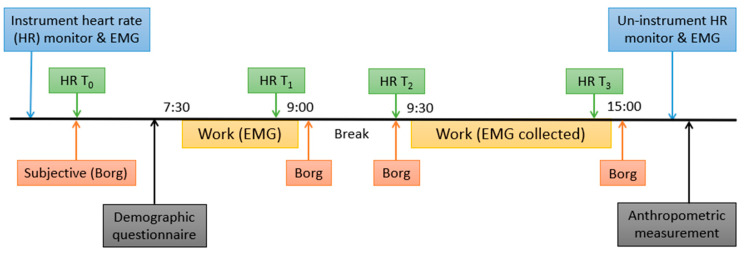
Study design showing the measurement time points for each measure.

These measurement time points are illustrated in a diagram, as shown in Figure 2.

### 2.4. Data Processing

#### 2.4.1. Metabolic Load: Percentage of Heart Rate Reserve

The raw heart rate data were filtered using a 5-point moving median to eliminate measurement artifacts. Then the means of the filtered heart rates for each period of interest, i.e., corresponding to the effort survey, were extracted. The time periods of interest for heart rate (T0, T1, T2, and T3) are also presented in Figure 2. Note that T0 and T2 were an exact match with the times of subjective measurement. Meanwhile, T1 and T3 for the heart rate measures were during the work period right before the break, rather than the heart rate measures while the participants were not working and answering the questionnaires.

The metabolic load was calculated in terms of the percentage of heart rate reserve (% HRR) during the work period based on Equations (1)–(3) where $HR_{max}$ is the maximal heart rate of an individual, $HR_{sit}$ is the heart rate measured while the participant was sitting for 10 min before starting work, $HR_{work}$ is the heart rate measured while the participant was working, and $HR_{rest}$ is the resting heart rate of an individual.

(1) $$HR_{max}=220-age$$

(2) $$HR_{rest}=HR_{sit}-10$$

(3) $$\%HRR=\frac{HR_{work}-HR_{rest}}{HR_{max}-HR_{rest}}$$

The % HRR was square-root transformed to meet the assumption of normality and verified by the Shapiro–Wilk test.

#### 2.4.2. Muscle Fatigue: EMG Median Power Frequency

The waw EMG signals were filtered with a 20–450 Hz bandpass filter. Then, errors and artifacts in the EMG data were diagnosed using a principal component analysis of several parameters, including the percentiles of the EMG amplitudes and the mean and median power frequencies, and then removed as described in our previous study [39]. By converting a time domain signal into a frequency domain, the MPF of the EMG signal was calculated for every 10 min. Then we conducted a linear regression of the MPF at each 10-min window on the time of the day for each trapezius side and each individual subject, based on Equation (4).

(4) $$MPF=b_{0}+b_{Time}Time$$

The slope of the time factor ($b_{Time}$) from Equation (4), which represented an increase or decrease in MPF over the work period, excluding the break, as indicated in the diagram in the Figure 2, was used in the analysis to identify the association between muscle fatigue and the changes over time in subject-reported local discomfort.

#### 2.4.3. Subjective Ratings: Borg RPE, Omni RPE, and Borg CR10

Unlike a previous study which recommended calibration to the maximum value of the scale [46], the effort surveys, including the Borg RPE, Omni RPE, and Borg CR10 scales, were analyzed at the specific time points in terms of an increase or decrease compared to the values at the beginning of the work shift (T0) to make the data interpretable.

### 2.5. Statistical Analysis

The relationship between direct and self-reported measures was investigated at both overall and local levels. For overall effort or full body exertion, the correlations between % HRR and Borg RPE, and the correlations between % HRR and Omni RPE were calculated. For local discomfort or muscle fatigue, the correlations between muscle fatigue (EMG MPF) and the Borg CR10 were calculated.

Initially, Pearson’s correlations between the subjective and direct measures were calculated. Then linear regressions were conducted to adjust for known confounders, that is, the harvesting method and the time of measurement for overall exertion, and the harvesting method and the side of trapezius (dominant or non-dominant) for local discomfort. Additionally, between the two subjective measures of overall effort, i.e., Borg RPE and Omni RPE, Spearman’s correlations were used as the non-parametric tests for their relationship.

Moreover, these measures were evaluated for the effect of harvesting method and work period. The effects on the % HRR were tested using ANOVA and the effects on the Borg RPE, Omni RPE, and Borg CR10 were tested using the Kruskal–Wallis test.

The level of statistical significance was set at 0.95, that is, we have 95% confidence in rejecting a null hypothesis, or a 5% probability of making an error in rejecting the null hypothesis while it was true. All the statistical analyses were conducted using R programming language. In particular, the “lm” function was used to run a linear regression analysis to find the correlation coefficients of the relationship between parameters and the “anova” function was used for extracting the significant levels of the effect. Additionally, for the nonparametric tests to show the effect of harvesting method and measurement time point on the subjective measures, the “dunn.test” package was used for post hoc pairwise comparisons using rank sums. All the graphics were made using the “ggplot2” package.

## 3. Results

### 3.1. Sociodemographic Characteristics of the Respondents

Twenty-four Hispanic male apple pickers participated in the study. Despite our efforts to include both genders in the participant recruitment, we could not find female volunteers to participate in the study since most of the workers were men. The participants’ ages were on average 28.4 years (range 18–47 years). Their experience as farmworkers harvesting tree fruits in the United States was on average 3.4 years (range 1–14 years).

### 3.2. Overall Effort: % HRR as Metabolic Load, Borg RPE, and Omni RPE

**Figure 3:**
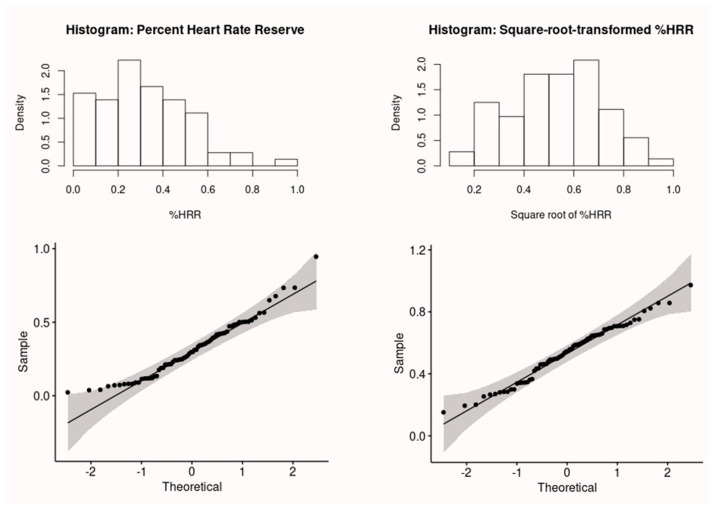
Histograms and Q–Q plots of the % HRR before and after square–root transformation.

The calculated % HRR was not normally distributed according to the *p*-value of 0.013 in the Shapiro–Wilk test for normality. After the % HRR was square-root transformed, the data became normally distributed, i.e., with a *p*-value of 0.48 in the normality test. Figure 3 shows the histograms and the Q–Q plots of the data before and after the transformation.

**Figure 4:**
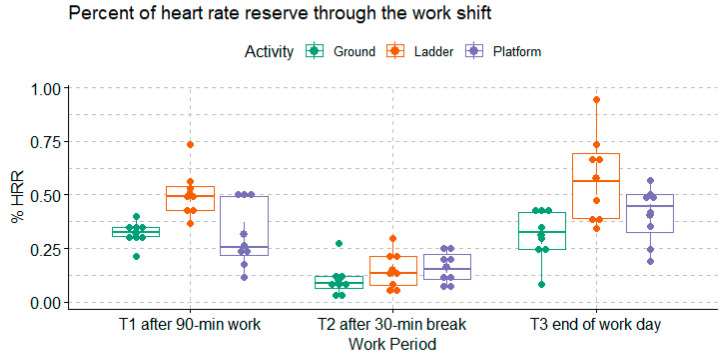
% HRR measured among each group of workers at each work period (n = 8 for each group).

The metabolic load, i.e., the % HRR, among each group of workers at each time of measurement is shown in Figure 4. In general, the % HRR values were between 0.15 and 0.75 after the participants had worked for 90 min (T1). Then the % HRR significantly dropped after a 30-min break (T2) and increased again at the end of the work shift (T3) (*p*-value < 0.0001). Overall, the % HRRs among the Ladder group were higher than the % HRR among the Ground and Platform workers (Tukey HSD *p*-value = 0.0001 for the comparison between the Ladder and Ground workers, and 0.009 for the comparison between the Ladder and Platform workers).

**Figure 5:**
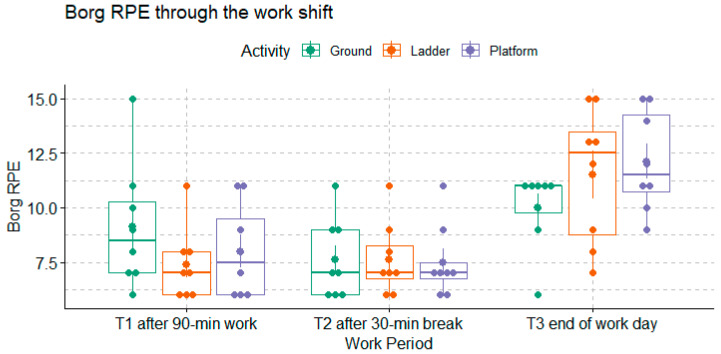
Borg RPE, a measure of overall exertion reported by each group of workers at each work period (n = 8 for each group).

As shown in Figure 5, relative to the Borg RPE ratings collected at the beginning of the work break (T0), there were no differences between the ratings collected at the beginning of the first break (T1) and after 30 min of rest (T2). The Borg RPE ratings collected at the end of the work shift (T3) were significantly greater than those measured at the beginning of the first break (T1) and those measured after the 30-min rest (T2) (Dunn’s test *p*-values = 0.0007 and 0.0001). Nevertheless, the Borg RPE was not significantly different across the harvesting methods (Dunn’s test *p*-value = 0.09).

**Figure 6:**
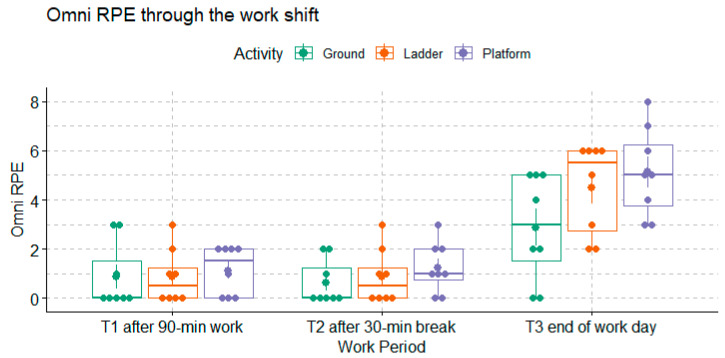
Omni RPE, a measure of overall exertion, reported by each group of workers at each work period (n = 8 for each group).

The differences in Omni RPE from the beginning of the work shift were also greater at the end of the work shift (T3) compared to the other time points (*p*-value < 0.0001), as shown in Figure 6. Comparing across workers’ groups, the Omni RPE was significantly higher among the Ground workers compared to the Platform and Ladder workers (Dunn’s test *p*-values = 0.04 and 0.02).

Finally, the Borg RPE and Omni RPE were found to be positively correlated with a Spearman’s correlation of 0.618.

### 3.3. Association between Metabolic Load and Subjective Overall Effort

Without adjusting for neither work period (T1, T2 and T3) nor harvesting method (Ground, Ladder, and Platform), the correlation coefficient between the % HRR and the Borg RPE was insignificant (*p*-value = 0.23). In contrast, the correlation coefficient between the % HRR and the Omni RPE was positive (*p*-value = 0.006).

In addition, when adjusted for the work period and the harvesting method, which had a significant effect on the % HRR, the regression coefficient between the % HRR and the Borg RPE became negative, with a *p*-value of 0.054. In the same way, the regression coefficient between the % HRR and the Omni RPE also became negative, with a *p*-value < 0.0001.

**Figure 7:**
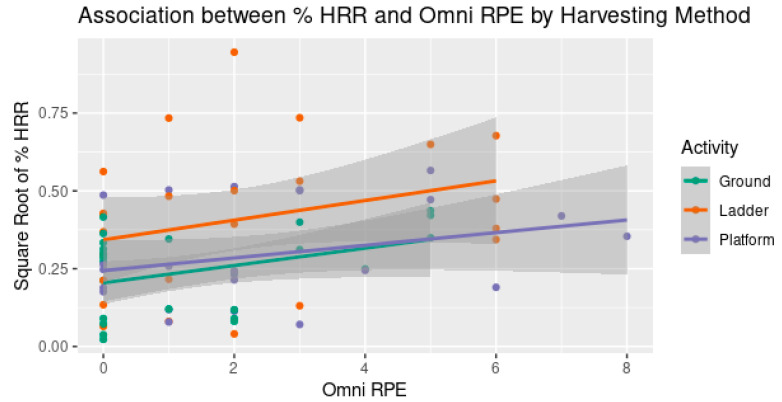
The statistically significant association between % HRR and Omni RPE in all groups when stratifying by harvesting method and combining all time points.

**Figure 8:**
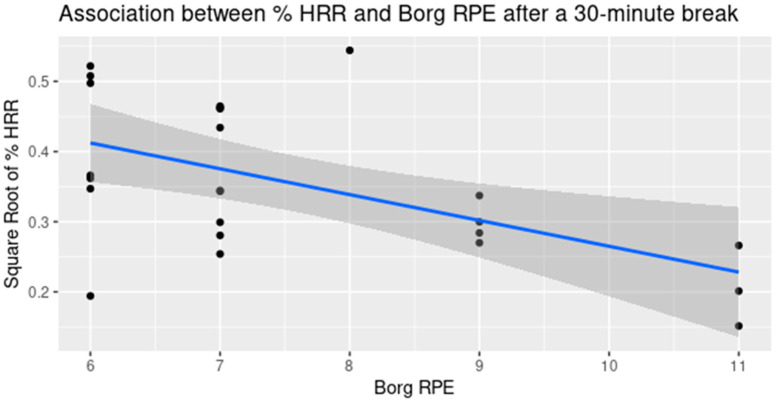
The statistically significant association between % HRR and Borg RPE after a 30-min break, combining all harvesting methods.

With a confounding effect, the analyses were further stratified by the harvesting method and by the work period. On the one hand, when the analysis was stratified by the harvesting method and the effect of the work period was adjusted, the correlations between the % HRR and the Borg RPE were no longer significant, meanwhile statistically significant or almost significant correlations between the % HRR and the Omni RPE were found in all the worker groups (*p*-value = 0.014, 0.015, and 0.086 for Ground, Ladder, and Platform groups, respectively), as shown in Figure 7. On the other hand, when stratified by the work period, i.e., the time point of the measurement, the correlations between the % HRR and the Borg RPE difference were found to be statistically significant only at T2 (*p*-value = 0.0041), as shown in Figure 8. Meanwhile, none of the correlation coefficients between the % HRR and the Omni RPE were statistically significant.

### 3.4. Local Discomfort: EMG MPF as Muscle Fatigue and Borg CR10

**Figure 9:**
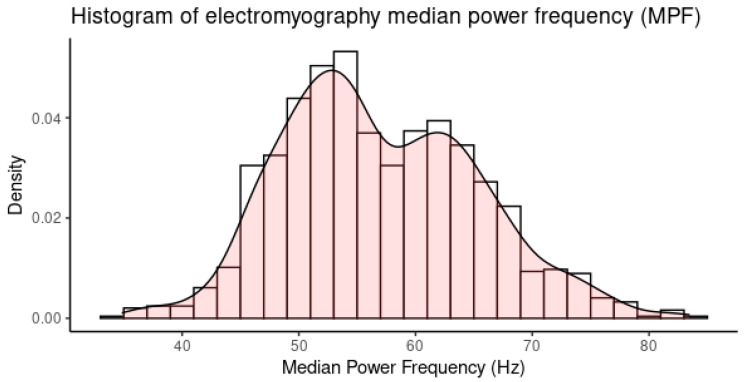
Histogram showing the bi-modal distribution of the EMG median power frequency.

**Figure 10:**
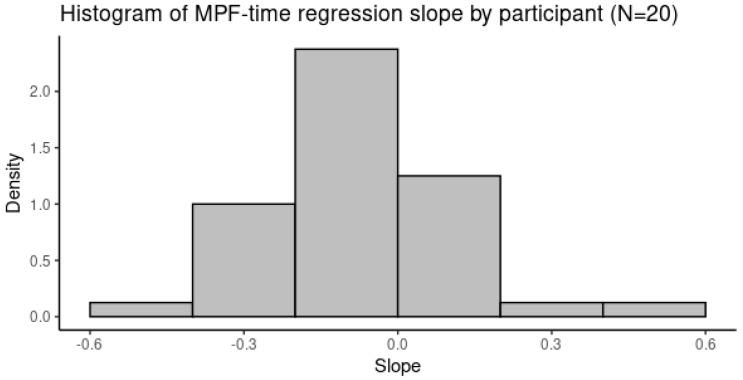
Histogram of the EMG median power frequency regression slope (b_Time).

The EMG MPF at the 10-min windows of all participants had a bi-modal distribution (Figure 9) due to the difference between the dominant and non-dominant muscle sides and the difference across harvesting methods as well as across the time of the day. These differences were adjusted using linear regression. After removing, i.e., adjusting for, the effects of muscle side ($b_{Side}$) and the effects of the participants ($b_{Subject}$) who were different across the harvesting methods, the slope of the time variable ($b_{Time}$) was used for analysis to find the correlation between the EMG MPF and the Borg CR10. Figure 10 shows the distribution of the $b_{Time}$ while the Shapiro–Wilk test indicated that the parameter could be considered as normally distributed (*p*-value = 0.059).

Muscle fatigue, i.e., the EMG MPF, decreased over time, as shown by the negative slope ($b_{Time}$ = −0.0056) in the regression Equation (4) (*p*-value < 0.0001). This is in accordance with the results for muscle activity from our previous study [39].

**Figure 11:**
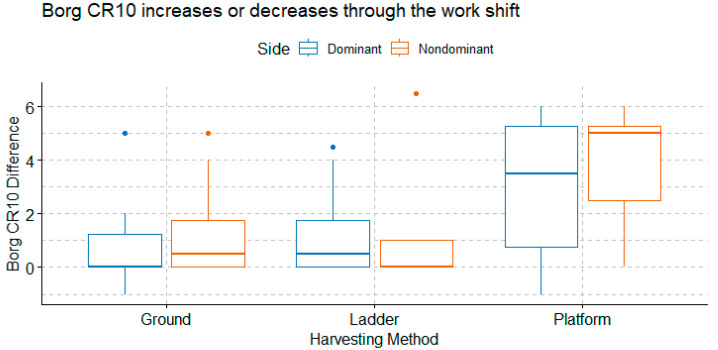
Borg CR10 difference between the beginning and the end of the work shift for harvesting method and muscle side.

The Borg CR10 difference between the beginning and the end of work shift for each harvesting method is shown in Figure 11. The increase in Borg CR10 from the beginning to the end of the work shift was higher in the Platform group than in the Ground and Ladder groups. According to the Kruskal–Wallis tests for nonparametric data, the harvesting method had a statistically significant effect on the Borg CR10 increase over time (*p*-value = 0.013), but the side of trapezius did not (*p*-value = 0.51).

### 3.5. Association between Muscle Fatigue and Subjective Local Discomfort

**Figure 12:**
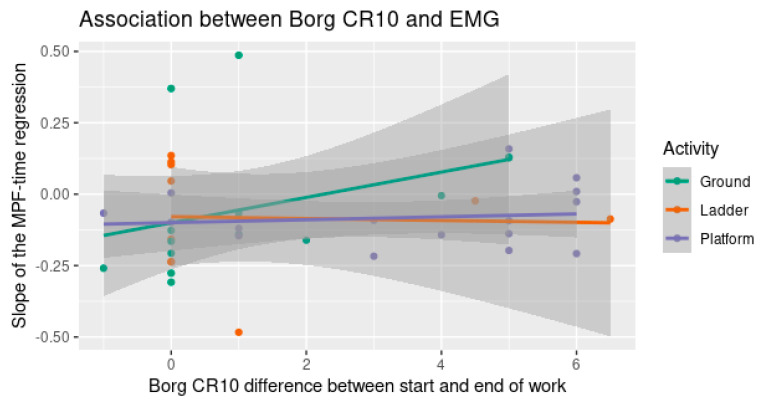
Scatter plot of the Borg CR10 difference on the *x*-axis and the $b_{Time}$ on the *y*-axis. All the correlations were nearly zero and the confidence intervals were large.

Regardless of whether we accounted for the muscle side, work period, or harvesting method, there was no correlation between the slope $b_{Time}$ in Equation (4), i.e., the increase or decrease in EMG mean power frequency over time and the Borg CR10 difference between the beginning and the end of the work shift. That is, there was no relationship between the EMG mean power frequency representing muscle fatigue and the Borg CR10 increases or decreases over the work period. Figure 12 shows a scatter plot of the association between the $b_{Time}$ on the *y*-axis and the Borg CR10 difference between the start and the end of the work shift on the *x*-axis.

## 4. Discussion

### 4.1. Interpretations and Implications from Negative or No Correlation

This study found significant correlations between the direct and subjective measures of overall effort when the analysis was adjusted for the harvesting method and the time of measurement; however, the directions of corrections were contradictory for the Borg RPE and Omni RPE. The negative correlations between the Borg RPE and % HRR suggest that the Borg RPE may not be useful as a subjective measure for this population, whereas the positive correlations between the Omni RPE and % HRR suggest that the Omni RPE could predict the outcomes of % HRR.

When stratifying by harvesting method, the significance level was still strong only when using the Omni RPE but not for the Borg RPE. This phenomenon was evident across all the worker groups. This finding of stronger correlations suggests that the Omni RPE with the pictures of tree fruit harvesters may be more useful than the Borg RPE with only verbal anchors on the scale from 6 to 20, which might not be comprehensible for farmworkers.

When stratifying by time periods in the work shift, a statistically significant relationship between the Borg RPE and % HRR was found at T2, i.e., after the lunch break, but not at T1 and T3, i.e., after the morning and afternoon work sessions. That is, when the effort was relatively light, the subjective Borg RPE responses could be meaningful. Otherwise, for periods of heavy workload, the use of the Borg RPE could not discern the effort levels. In other words, the Borg RPE was not interpretable in this population and might not be useful to assess recovery from rest in place of a direct measurement. Additionally, when stratifying by the time period in the work shift, the relationship between the Omni RPE and % HRR became statistically insignificant. This finding contradicts the stratification by harvesting methods. As a result, the Omni RPE with the pictures of tree fruit harvesters carrying a harvesting bag at different phases of tiredness (Figure 1) may still not be robust and should be improved.

There was no significant correlation between the direct and subjective measures of local discomfort. In other words, the Borg CR10 scales for local body parts, particularly the shoulders, were not representative of the muscle fatigue as directly measured and characterized by EMG. Thus, the use of the Borg CR10 to assess local body fatigue is not recommended for this population.

Despite being translated and adapted to the culture, the subjective effort surveys, namely the Omni RPE, Borg RPE, and Borg CR10, may not be suitable for ergonomic assessments among Hispanic fruit pickers, especially in this case when the physical workloads were extreme. Therefore, they could not fully replace the directly measured physiological outcomes such as metabolic load or muscle fatigue.

### 4.2. Comparisons to Previous Studies and Directions for Future Work

This study indicated the unsuitability of subjective scales for ergonomic assessment in fruit harvesting tasks undertaken by Hispanic migrant workers, compared to the uses of cardiac measures and the muscle fatigue of the trapezius. However, the findings from this study that the subjective scales were more sensitive for a light workload (T2) is opposite to a previous work, that is, while the Borg scale could detect a major change in task difficulty, it was found unsuitable to identify minor changes in task difficulty and discomfort, in contrast to the capability of EMG at the biceps brachii and triceps brachii [56]. On the contrary, the Borg CR10 was found to be more sensitive to a light load than the EMG MPF; that is, in a laboratory study using the EMG MPF of the trapezius and the Borg CR10 during arm abduction, there was a strong negative correlation between the MPF and the CR10 scores at a heavy load while the MPF did not change at a low load [57]. Above all, even though this study found increases over time in both EMG MPF and Borg CR10, we did not address whether a direct measure of muscle fatigue, such as EMG, or a subjective discomfort response, such as the Borg scales, could provide a better ergonomic assessment.

Newly developed subjective rating scales such as the Omni RPE may be used in certain contexts. This subjective measurement could be used as a complement to corresponding direct measurements rather than as a standalone tool. Even though a previous study found that the Omni RPE in a pictorial face format was correlated with heart rate and respiratory rate measurements in both men and women [58], the Omni RPE alone was not distinguishable across different walking and running loads in children, whereas oxygen consumption was [59]. Alternatively, we propose a combination of illustrations of farmworkers carrying apple bags and faces representing emotions in the Omni RPE.

### 4.3. Study Limitations

A number of systematic biases in this study should be mentioned. Firstly, the presence of researchers in the field might have altered the way the participants worked; thus, the directly measured outcomes for muscle fatigue or metabolic load may have been affected. Secondly, the administration of the Borg and Omni questionnaires could have interrupted the workers’ lunch break. It is possible that some workers could have answered the questions quickly rather than attentively.

Moreover, there was a limitation associated with the heart rate measurement. In an ideal situation, the resting heart rate should be measured in a recumbent position, but this was not possible in the orchard setting. However, in this study, the heart rates were measured during a quiet period in a sitting position prior to the start of the work shift, and then the measured values were subtracted by 10 as per Equation (2), similarly to our previous work [24]; in other words, the resting heart rate was obtained by approximation rather than exact measurement.

Furthermore, there were challenges in the EMG measurements in the field due to the perspiration of workers and the physical contact between the electrodes and the apple bag strap or the ladder. Anomalies in the EMG data were detected and removed with a new algorithm to retain the muscle activity signal [39] but there was still some data loss. Future studies should instead find a way to detect the anomalies in real time during the data collection, which could prevent data loss more effectively compared to logging the data to examine later at the end of the work shift as in this study.

Above all, these limitations could be anticipated prior to the study. On the one hand, part of the systematic bias during the data collection was not able to be fully eliminated due to the nature of the fieldwork. That is, firstly, the researchers were present and might have indirectly influenced how the farmworkers behave, and, secondly, the quiet sitting period for the resting heart rate measurement was difficult to be ensured. On the other hand, the data loss due to the EMG connection was mitigated by the development of the algorithms to remove the errors and retain only the meaningful signals.

## 5. Conclusions

This study examined whether there was a relationship between subjective and direct measures of overall cardiovascular load and local muscle fatigue among Hispanic migrant farmworkers harvesting apples in North America. The Borg RPE and Borg CR10 were translated into Spanish, and an Omni RPE with pictures was created for this specific population. The Borg RPE and Omni RPE results were compared to the metabolic load derived from heart rate data, which represented the overall physical exertion. This study found some strong negative correlations between the direct and subjective measures: the % HRR and Borg RPE after the workers took a short break but not after they performed hard work, and the % HRR and Omni RPE when stratifying by harvesting methods. The Borg CR10, which was expected to indicate local discomfort, was compared to muscle fatigue as characterized by EMG at the trapezius. Unlike the results of the correlations between the RPEs and the cardiovascular load, there was no significant correlation between the Borg CR10 and EMG. All things considered, for the Hispanic farmworker population, direct measures of ergonomic exposure could not be replaced by subjective measures according to this study. If only subjective measures are possible in field assessments, the results will have to be interpreted with caution. If necessary, the Omni RPE, i.e., the scales accompanied by pictures, would be a better option than the Borg RPE or Borg CR-10, which only have verbal anchors and were developed during different activities performed by different populations.

## Data Availability

The data presented in this study are openly available in a public code repository and can be found at https://github.com/ornwipa/subjective_objective/tree/master/data (accessed on 1 February 2023).
